# Development and Psychometric Evaluation of the Perceived Diet Quality Questionnaire

**DOI:** 10.3390/nu18203323

**Published:** 2026-10-10

**Authors:** Jessica Thomson, Alicia Landry, Tameka Walls

**Affiliations:** 1 Delta Human Nutrition Research Program, USDA Agricultural Research Service, 5832 I-49 N Service Road Suite 1, Opelousas, LA 70570, USA; 2 School of Kinesiology and Nutrition, The University of Southern Mississippi, 118 College Drive, Hattiesburg, MS 39406, USA

**Keywords:** perceived diet quality, focus groups, factor analysis, reliability, questionnaire development, psychometric evaluation, nutrition practices, nutrition attitudes

## Abstract

Background/Objectives: Perceived diet quality (PDQ), measured with a single question, has been linked to better cardiometabolic health indicators. However, no published comprehensive tool to measure PDQ exists. Therefore, the objective of this study was to describe the development and initial psychometric evaluation of a novel tool to measure perceived diet quality (PDQ). Methods: Development included focus groups (*n* = 27) and thematic analysis. Psychometric evaluation included expert reviews (*n* = 3), cognitive interviews (*n* = 13), and online cross-sectional nonexperimental survey (*n* = 605). Eleven items—with 3 addressing perceived diet quality based on qualitative, graded, and numeric ratings, and satisfaction with diet healthfulness, balanced meals, meal planning/preparation, nutrition knowledge, home cooking vs. eating out/takeout, food label use, difficulty eating healthy, and guilt/regret about food choices comprising the other items. Analysis included exploratory factor analysis, concurrent and convergent validity, and construct reliability. Results: Seven of the 11 variables were retained for final factor solution. Mean (SD) of PDQ Scale, possible score range 6.5–35 points, was 22.6 (5.4). Construct reliability was demonstrated by McDonald’s omega of 0.91. Concurrent validity was demonstrated by correlation of 0.74 between single qualitative PDQ item (current assessment method) and PDQ Scale. Weak to moderate convergent validity was demonstrated by correlations of 0.31 and 0.51 between the PDQ Scale and diet quality scores measured using the Healthy Eating Index and Global Dietary Recommendations, respectively. Correlations were generally significantly higher than correlations with single qualitative PDQ item. Conclusions: The PDQ Scale may be better measure of self-assessed diet quality than the current single qualitative item.

## 1. Introduction

The United States healthcare system continues to face escalating costs driven by chronic diseases with nutritional etiologies. Adherence to a high-quality diet is associated with reduced risk of chronic diseases such as type 2 diabetes, cardiovascular disease, and cancer, all of which are diseases with nutritional etiologies [1,2,3]. Personalized nutrition is a promising field with the goal of improving diet quality and health outcomes by providing recommendations that are more personally relevant and meaningful. This personalized approach, which integrates an individual’s unique characteristics, may help alleviate some healthcare costs and overall chronic disease burden. However, for personalized nutrition to realize its full potential, individuals need to have an accurate perception of the quality of their diet. Accordingly, there is a critical need for tools that can validly and reliably assess perceived diet quality. In the absence of such tools, the conduction of studies designed to identify interventions or approaches that can improve the accuracy of self-assessed diet quality is limited.

Currently, a single-item tool is used to measure perceived diet quality (PDQ), and it has been promoted as a simple and rapid screening method for identifying individuals with poor diet quality and/or at risk for poor cardiometabolic health [4,5,6,7]. Perceived diet quality has been positively associated with actual or measured diet quality in United States adults [6,8,9,10,11], and higher PDQ has been linked to better cardiometabolic health indicators [6], while lower PDQ has been linked with higher odds of cardiometabolic risk [7]. Further, higher PDQ was associated with more beneficial attitudes towards nutrition such as greater interest in consuming a healthy diet and recognition of the importance of nutrients and food groups for optimal health [4]. However, association does not guarantee accuracy and, unfortunately, adults in the United States do not accurately perceive their diets, most often overrating the quality of their diets [12,13]. Almost one fourth of US adults with poor diet quality perceive their diet as healthy [12]. This misperception is troubling as it may hinder motivation to change and can undermine public health campaigns designed to promote healthful dietary habits. Part of the misalignment between actual and perceived diet quality may be due to how PDQ is assessed. Currently, PDQ is measured with a single question asking how healthy one’s general or overall diet is. The concept of healthy eating is complex, with different and diverse interpretations depending on personal, social, cultural and environmental contexts [14]. Thus, it is unlikely that a single question can adequately measure the complex concept of perceived diet quality.

At present, no published comprehensive tool to measure PDQ exists. A tool with evidence of validity and reliability is needed to conduct systematic examinations of PDQ and its associations with dietary intake and other health outcomes. To bridge this gap, we developed a novel tool to measure PDQ, tested it in a sample of individuals from three states, and examined its internal factor structure. This article describes the development and initial psychometric evaluation of the Perceived Diet Quality Questionnaire and Scale, including results of exploratory factor analysis.

## 2. Materials and Methods

**Figure 1:**
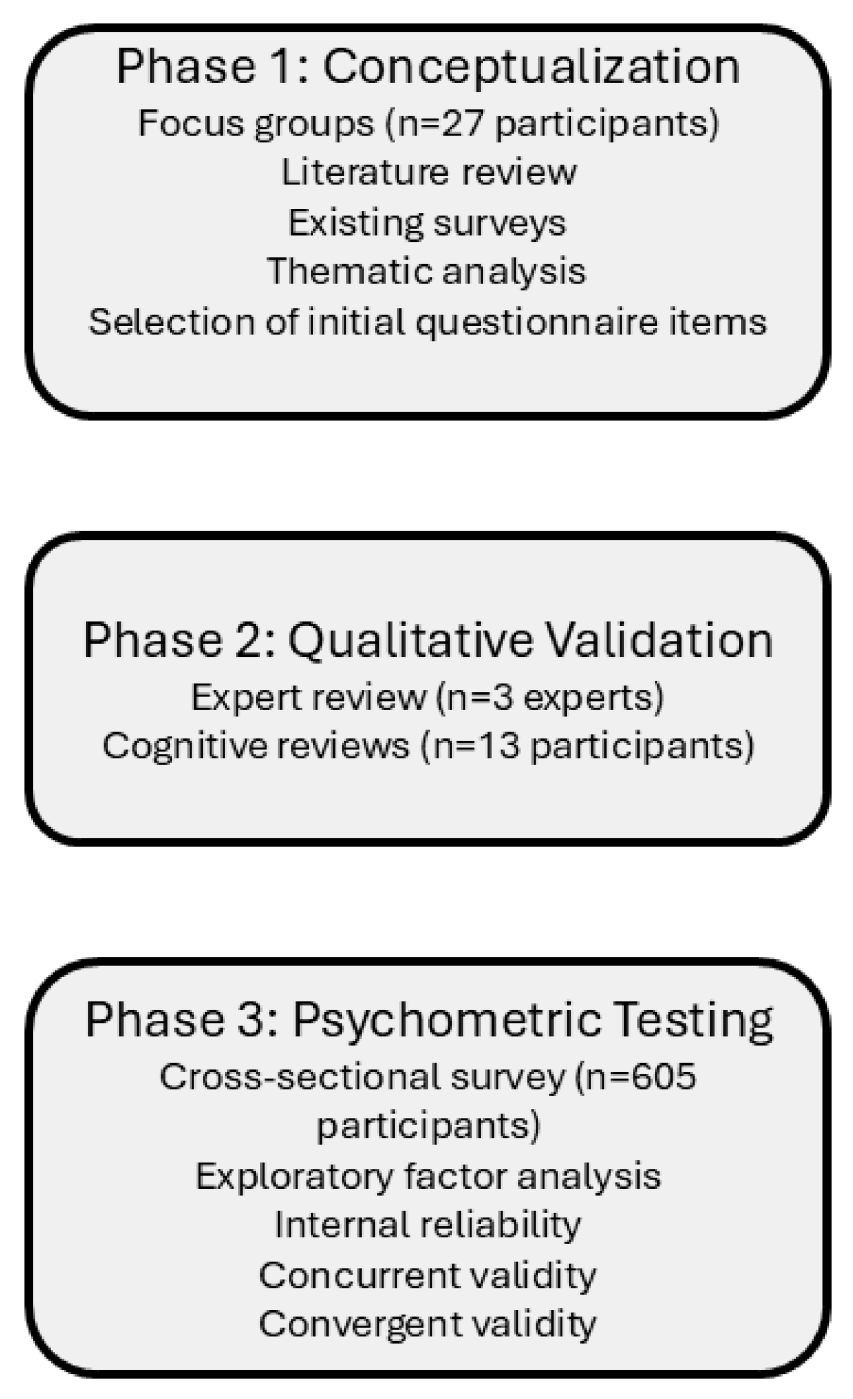
Flowchart of project design phases.

Development of the PDQ Questionnaire was conducted in sequential stages (Figure 1). First, we conducted focus groups to determine what individuals think about when asked to rate their diet quality (8 focus groups with total *n* = 27 participants) [15]. Second, based on thematic analysis of the focus group discussions [15], existing surveys, and a literature review of healthy eating, we selected items for inclusion on the PDQ Questionnaire. We embedded items for the PDQ Questionnaire within a larger survey that contained a question about general health, the Diet Quality Questionnaire from the Global Diet Quality Project [16,17], questions about dietary plans/restrictions, reasons for difficulty eating healthy, and a set of demographic items. Items specific to the PDQ Questionnaire included self-assessed diet quality (qualitative, graded, and numeric ratings), satisfaction with healthfulness of diet, balanced meals, meal planning/preparation, home cooking vs. eating out/takeout, nutrition knowledge, food label use, difficulty eating healthy, and guilt/regret about food choices. Item wording, responses, and sources are presented in Table 1. To determine if placement affected responses, we asked the 3 self-assessed diet quality items twice, once at the beginning of the questionnaire and again at the end of the questionnaire before the demographic items.

Next, content validity of the PDQ Questionnaire was supported through review by subject matter experts who evaluated the relevance of the items. The 3 experts were university colleagues selected for their expertise in nutrition (registered dietitians) and experience conducting or managing nutrition research studies (including survey administration). We established face validity of the PDQ Questionnaire through cognitive interviews with community individuals who were selected from lists of small-scale farmers and school food service directors, groups with which we had previously worked. To increase chances of participation, selection preference was given to those recommended to us by members of the focus groups or who resided or worked in Mississippi communities in which we previously conducted research. We conducted 3 separate cognitive interviews with convenience samples of 2–6 adults (≥18 years of age; total sample size = 13). A trained and experienced researcher moderated interviews virtually via Zoom. Examples of probes used in the interviews included asking if questions were hard to answer and if response options did not feel appropriate. Participants provided electronic informed consent prior to participation in the cognitive interviews and received a $25 e-gift card for their time. The study was reviewed and approved through an expedited review process by the Institutional Review Board of Delta State University (protocol no. 2024-013, 5 October 2023; protocol no. 2025-048, 25 March 2025).

Finally, we implemented the PDQ Study, consisting of a cross-sectional nonexperimental survey. Based on a priori sample size analysis hypothesizing that the new tool would consist of 2 factors with 3 indicators per factor, factor loading magnitudes of 0.5, factor correlations of 0.3, and 20% missing data, a sample size of 425 was sufficient for factor analysis with a power of 0.80. We recruited study participants from convenience samples of individuals residing in the states of Arkansas, Louisiana, and Mississippi. While individuals with lower socioeconomic status were of interest to us, it was not our intent to only include such individuals in the study. Using the survey data, we conducted exploratory factor analysis of the PDQ Questionnaire items and established concurrent and convergent validity. Data collection occurred via Snap Online (Snap Surveys Ltd., Portsmouth, NH, USA) from April to September 2025. We recruited participants via onsite interactions, word of mouth, and primarily through flyers posted at various organizations, including food banks/pantries/networks, health care clinics/centers, nonprofit community programs, and churches. Flyers contained a quick response code to access the recruitment survey. Following completion of the recruitment survey, we enrolled eligible individuals into the study by email, inviting them to complete the PDQ Questionnaire using an embedded link. Upon completion of the PDQ Questionnaire, we sent participants another email inviting them to complete a Diet ID™ (eating pattern) assessment using a personalized link embedded in the email. Participants provided electronic informed consent prior to participation in online surveys and received $40 e-gift cards for their time. The PDQ Study was reviewed and approved through an expedited review process by the Delta State University’s Institutional Review Board (protocol 2025-048).

We measured PDQ using 3 items based on qualitative, graded, and numeric ratings. The qualitative question was “In general, how healthy is your diet?” with 5 responses including excellent, very good, good, fair, and poor. The graded question was “Think of letter grades, like in school, how would you ‘grade’ the quality (healthfulness) of your diet?” with 5 responses including A, B, C, D, and F. The numeric question was “How would you rate the quality (healthfulness) of your diet? Enter a number between 1 (horrible) to 10 (perfect).” All items were coded such that higher ratings corresponded to higher numbers (e.g., excellent and A = 5). The items were selected based on results from the qualitative study using focus groups to determine what individuals think about when asked to rate the quality of their diet [15].

We measured satisfaction with healthfulness of diet, balanced meals, meal planning/preparation, home cooking vs. eating out/takeout, nutrition knowledge, food label use, difficulty eating healthy, and guilt/regret about food choices with a single question each and with 5 responses for each, except for difficulty eating healthy. Responses for satisfaction with healthfulness of diet included very satisfied, satisfied, neither satisfied nor dissatisfied, dissatisfied, and very dissatisfied. Responses for balanced meals, meal planning/preparation, food label use, and guilt/regret about food choices included always, often, sometimes, rarely, and never. Responses for home cooking vs. eating out/takeout included all meals cooked at home, most meals cooked at home, about the same between meals cooked at home and eating out/takeout, most meals eaten out/takeout, and all meals eaten out/takeout. Responses for nutrition knowledge included strongly agree, agree, neither agree nor disagree, disagree, and strongly disagree. All items were coded such that more positive responses corresponded to higher numbers [e.g., very satisfied, always (never for guilt/regret), all meals cooked at home, and strongly agree = 5]. For the item difficulty eating healthy, the 3 responses (and coding) included yes—always (1), yes—sometimes (2), and no (3).

We captured dietary intake and diet quality using 2 methods. The first method was based on the Diet Quality Questionnaire (DQQ) from the Global Diet Quality Project [16,17]. The DQQ is a validated, standardized tool designed to collect indicators of dietary adequacy, consumption of 5 recommended food groups, protection of health against and risk of noncommunicable diseases, and alignment with global dietary recommendations [16]. The DQQ is composed of yes/no questions about 29 food groups consumed during the previous day or night [17]. Based on responses to the 29 food groups, 5 indicator scores are computed—Dietary Diversity, All 5, Noncommunicable Diseases Protect, Noncommunicable Diseases Risk, and Global Dietary Recommendations (GDRs). Only the GDR score (range 0–18) was used in the current study as an indicator of overall diet quality with higher scores indicative of meeting more healthful dietary recommendations. While DQQ is designed to assess population-level diet quality [16], it was used in this study at an individual level, although not as a diagnostic or clinical tool. The DQQ was recreated as an online electronic questionnaire using Snap XMP Desktop software version 12.22 (Snap Surveys Ltd., Porstmouth, NH, USA). All data were collected and stored on the Snap WebHost, an online mobile and secure survey management system.

The second dietary collection method was based on the Diet Quality Photo Navigation^®^ technique via the Diet ID™ tool. The photo navigation technique is a patented image-based algorithm that provides estimates of nutrient intake based on a series of food images [21,22]. Diet ID™ provides nutrient estimations for energy intake, macronutrients, and micronutrients, including phytonutrients and other bioactive compounds, based on the Nutrition Data System for Research food database [21,22]. It is designed to measure usual dietary patterns over the last 30 days. Participants completed a Diet ID™ assessment using a personalized link sent to them via email. After clicking on the link, participants completed a set of screening questions to identify select food group consumption, such as vegetarian or gluten-free dietary patterns. Participants then chose between 2 images containing a variety of food items to identify general types of food consumed. Subsequently, participants chose between 2 additional images containing varying types of the same food, such as low-fat vs. full-fat dairy products. The process continued until the application identified participants’ typical eating pattern. Foods from the final image were quantified for nutrient analysis by the Diet ID™ algorithm. In addition to specific nutrient output, 2020 Healthy Eating Index (HEI) scores, an a priori diet quality measure, were computed. The HEI measures alignment with the Dietary Guidelines for Americans [23], ranges from 0 to 100 points, and is composed of 9 adequacy and 4 moderation components [24]. Details regarding HEI scoring standards can be found on the Food and Nutrition Administration website [25]. Higher HEI scores are indicative of a more healthful diet. We instructed participants to take the Diet ID™ assessment only once. For those who completed multiple assessments, we used the first complete assessment for analysis. For the purposes of this study, we refer to GDR and HEI scores as “measured diet quality.”

Demographic characteristics included age (years), sex (male or female), relationship status, education level, work status, ethnicity (yes or no Hispanic/Latino/Spanish), race, and yearly household income. The 7 age categories were 18–19, 20–29, 30–39, 40–49, 50–59, 60–69, and ≥70 years. The 6 relationship categories were married, widowed, divorced, separated, never married, and living with partner. The 6 education categories were ≤some high school, high school diploma, some college, associate or technical degree, Bachelor’s degree, and graduate or professional degree. The 6 work categories were employed full time (≥30 h/week), employed part time (<30 h/week), not employed and looking for work, not employed and not looking for work, retired, and disabled and not able to work. The 6 race categories were American Indian or Alaska Native, Asian, Black or African American, Native Hawaiian or Pacific Islander, White, and other. The 12 income categories started with ≤$25,000, increased by $10,000 per category, and ended with the last category of ≥$125,001. For analytic purposes, categories were combined as follows: the first 2 and last 2 age categories combined as 18–29 and ≥60 years, respectively; relationship status combined as married/living with partner and divorced/widowed/separated; the first 2 and last 2 education categories combined as ≤high school diploma, and ≥Bachelor’s degree, respectively; the last 3 work categories combined as not employed; ethnicity and race combined as non-Hispanic Black, non-Hispanic White, and other/multiracial; and the last 8 income categories combined as ≥$55,001. All the demographic items included a “Prefer not to say” response that was coded as missing.

We used SAS^®^, version 9.4 (SAS Institute, Inc., Cary, NC, USA) to conduct analyses with statistical significance set at *p* < 0.05. We computed descriptive statistics for participant characteristics and items included in the factor analysis. We computed the Kaiser–Meyer–Olkin test to determine sampling adequacy for factor analysis (Kaiser–Meyer–Olkin > 0.60 for the total set of items and >0.50 for individual items) [26]. We conducted exploratory factor analysis of the PDQ Questionnaire using the Proc Factor procedure with initial prior communality estimates set to squared multiple correlations. We used iterated principal factor analysis as the extraction method on polychoric correlation matrices for ordinal data with an oblique (promax) rotation to allow for correlated factors. We based factor retention on several criteria including visual scree plot (based on “elbow bend” in plot) [27], parallel analysis with 1000 random samples [28], minimum average partial [29], and interpretability [30]. For parallel analysis, factors with eigenvalues in the observed data that exceed those from the simulated data are retained [31]. For minimum average partial, the number of factors retained is based on the smallest value associated with the lowest number of factors [31]. We retained items based on several criteria including pattern coefficients (factor loadings ≥ 0.5) [32], item communality (≥0.4), no cross-loadings ≥ 0.32 on alternative factor and/or difference ≥ 0.20 between primary and alternative factor loadings [33,34], number of items per factor (≥4 items) [35], and interpretability. We assessed the internal reliability of the final scale using McDonald’s omega [36] with a value ≥ 0.70 considered evidence of good reliability [32]. The single qualitative PDQ question was used as the reference standard because it is the most established and frequently used method to measure self-assessed PDQ. Hence, to establish concurrent validity, we assessed Spearman’s correlation between the single qualitative PDQ item and the final factor solution. We represented the final factor solution as a sum score of the retained items and labeled it the PDQ Scale. For the purposes of this study, no missing items were allowed when calculating the PDQ Scale. To keep all weights equal, we multiplied the PDQ numeric rating by 0.5 because its range was 1–10 (vs. 1–5 for the other 6 items). Because the single qualitative item was part of the final factor solution, we computed correlations for the full PDQ Scale and for the reduced PDQ Scale without the qualitative item to avoid inflated correlations. We assessed convergent validity using Spearman’s correlations between the HEI and GDR scores and the PDQ Scale. Finally, to determine if the PDQ Scale performed better than the single qualitative PDQ item, we computed Spearman’s correlations between the total HEI and GDR score and the single qualitative PDQ item and compared them to the PDQ Scale correlations using Steiger’s Z test for dependent correlations. We conducted analyses separately for the first and second set of diet quality indices and observations with missing data were excluded listwise from the analyses.

## 3. Results

Expert review of the PDQ Questionnaire resulted in minor wording and placement changes to some questions, thereby supporting content validity of the new tool. Although this was not a formal, systematic content review, their expert feedback provided meaningful confirmation that the items appropriately reflected the construct of perceived diet quality. Based on feedback from the cognitive interviews, we modified the PDQ numeric rating item to allow for decimal (e.g., 8.5) vs. whole number responses, added response categories to the reasons for finding it hard to eat healthy (e.g., dislike cooking or no desire to prepare foods), and made additional minor wording changes (e.g., price of healthy foods revised to cost of healthy foods). Thus, face validity was established.

Of the 1337 recruitment surveys completed for the PDQ Study, 962 (72%) individuals were enrolled in the study. Reasons for non-enrollment included duplicate name, email address, or telephone number (*n* = 273; 73%); suspicious or invalid contact information (*n* = 57; 15%); no response to telephone call verification (*n* = 33; 9%); study closed (*n* = 10; 3%); declined participation (*n* = 1; <1%); and ineligibility (*n* = 1; <1%). Of the 962 individuals enrolled in the study, 610 (63%) completed the PDQ Questionnaire; however, five questionnaires were excluded due to mismatch between enrollment and PDQ Questionnaire participant name (*n* = 3) and duplicate questionnaires completed by same participant using different email addresses (*n* = 2). Of the 605 valid participants who completed the PDQ Questionnaire, 591 (98%) also completed the Diet ID™ assessment. We present participant characteristics (*N* = 605) in Table 2. The majority of the participants were between 20 and 49 years of age (68%), female (71%), employed full time (≥30 h/week; 52%), non-Hispanic Black (67%), and had an income ≤ $45,000/year (52%). Approximately one third of participants were married and never married. One fourth of participants had a high school diploma while one third had a Bachelor’s degree or higher.

The overall model Kaiser–Meyer–Olkin value was 0.91, indicating sampling was adequate. Individual variable Kaiser–Meyer–Olkin values were ≥0.90 except for having difficulty eating healthy (0.81) and guilt/regret about food choices (0.58). The Kaiser–Guttman rule, minimum average partial (one-factor and two-factor squared partials = 0.029 and 0.042 first set; 0.024 and 0.035 second set), and interpretability suggested a one-factor structure while the scree plot and parallel analysis (first set observed eigenvalues = 5.4 and 1.3, simulated values = 1.3 and 1.2, respectively; second set observed = 5.5 and 1.3, simulated = 1.3 and 1.2, respectively) suggested a two-factor structure. For the final solution, we retained a single factor composed of 7 of the original 11 items. The seven items included qualitative, graded, and numeric diet quality, satisfaction with healthfulness of diet, balanced meals, meal planning/preparation, and nutrition knowledge. Because the second factor included only two items with one item that cross-loaded on the first factor (difficulty eating healthy and guilt/regret about food choices), we did not retain it. We present pattern coefficients and communality values for the original and final factor matrices in Table 3. Pattern coefficients for the retained items were ≥0.62 and item communalities were ≥0.38. While the communality for nutrition knowledge was <0.40 for the first set of indices, it was 0.43 for the second set of indices and thus retained in the final factor solution. McDonald’s omega was 0.91 and 0.92 for the first and second set of indices, demonstrating strong internal reliability [37].

**Figure 2:**
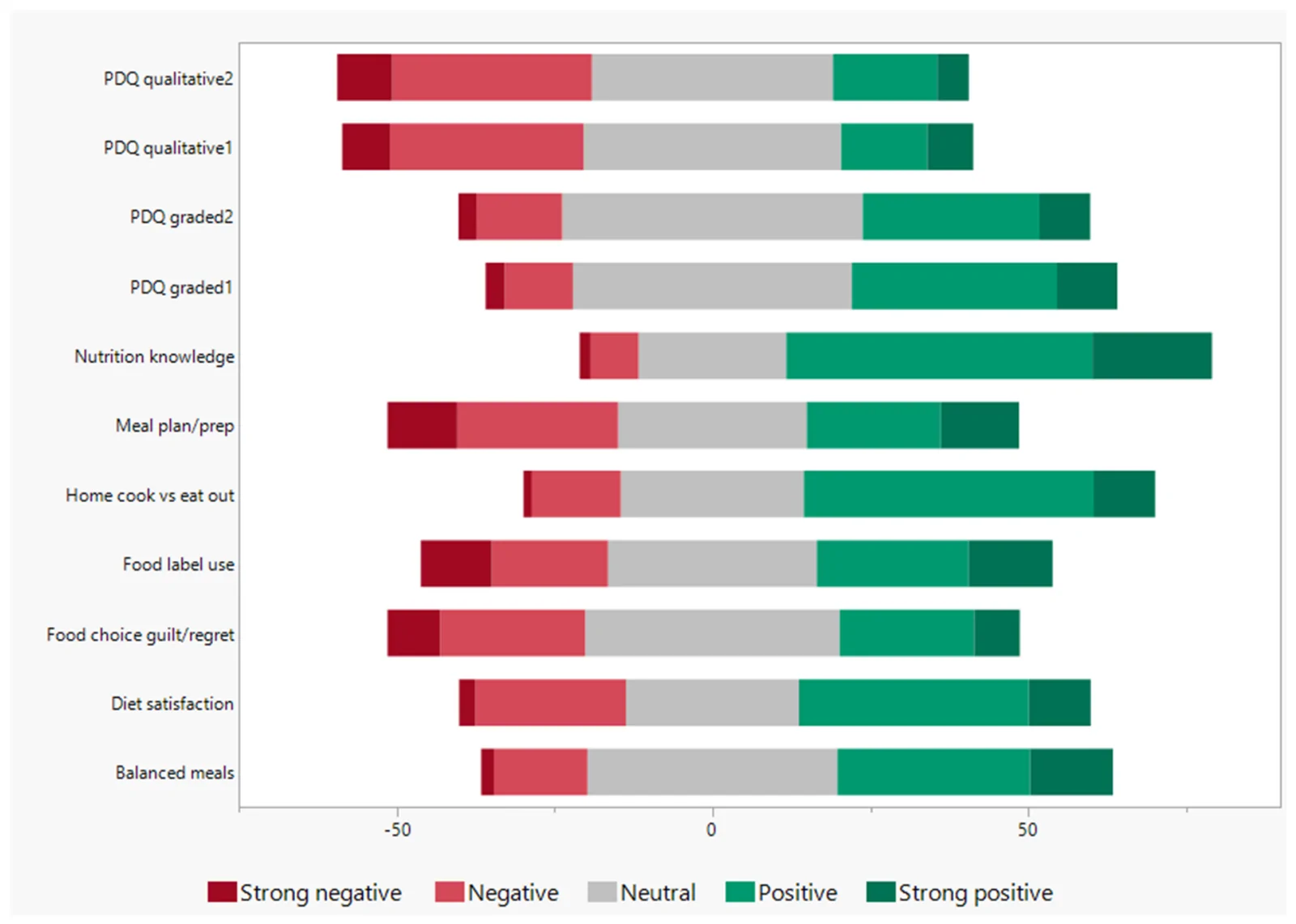
Response percentages (%) of the Perceived Diet Quality Questionnaire items. Item response categories from very negative (−% on *x*-axis) to very positive (+% on *x*-axis) are as follows. Perceived diet quality (PDQ) qualitative1 and qualitative2: poor, fair, good, very good, excellent. PDQ graded1 and graded2: F, D, C, B, A. Nutrition knowledge: strongly disagree, disagree, neither disagree nor agree, agree, strongly agree. Meal plan/prep, food label use, balanced meals: never, rarely sometimes, often, always. Home cook vs. eat out: all meals eaten out/takeout, most meals eaten out/takeout, about the same between meals cooked at home and eating out/takeout, most meals cooked at home, all meals cooked at home. Food choice guilt/regret: always, often, sometimes, rarely, never. Diet satisfaction: very dissatisfied, dissatisfied, neither dissatisfied nor satisfied, satisfied, very satisfied.

We present response frequencies for the PDQ Questionnaire items in Figure 2. For the PDQ specific items, the most frequent responses were good for both the first and second sets of the qualitative item (41% and 38%) and C for both the first and second sets of the graded item (44% and 48%). Mean values were 6.1 and 5.8 for the first and second sets of the numeric item. Response frequencies increased from strongly disagree to agree for good knowledge of nutrition (2–49%), increased from very dissatisfied to satisfied for satisfaction of diet healthfulness (3–36%), and increased from all meals eaten out/takeout to most meals cooked at home for the item home cooking vs. eating out/takeout (1–46%). The most frequent response for meal planning/preparation, food label use, food choice guilt/regret, and balanced meals was sometimes (30%, 33%, 40%, and 40%, respectively). For difficulty eating healthy, half (50%) of participants responded yes—sometimes with approximately equal percentages (25%) for yes—always and no responses.

We created the PDQ Scale by summing the scores of the seven retained items resulting in a possible score range of 6.5–35 points. For simplicity and robustness across samples (i.e., avoid overfitting), we chose not to weight items—with the exception of the PDQ numeric rating (multiplied by 0.5), to keep its influence equal to the other items [38]. Scale mean values (SD) were 22.6 (5.4) and 22.3 (5.4) for the first (*n* = 531) and second (*n* = 557) sets of indices. Correlations between the full PDQ Scale and the single qualitative PDQ item were strong [0.82 (*n* = 531) and 0.83 (*n* = 557); *ps* < 0.001] for the first and second sets, supporting sufficient concurrent validity for the PDQ Scale [38]. Correlations between the reduced PDQ Scale (single qualitative item removed) and the single qualitative PDQ item were also strong (0.74 and 0.76; *ps* < 0.0001) for the first and second sets. Correlations between the PDQ Scale and HEI were weak for the first and second sets [0.33 (*n* = 521) and 0.31 (*n* = 546); *ps* < 0.001], while correlations between the PDQ Scale and GDR were moderate for the first and second sets [0.51 (*n* = 459) and 0.51 (*n* = 481); *ps* < 0.001], suggesting less than optimal convergent validity for the PDQ Scale [38]. Correlations between the single qualitative PDQ item and HEI were weak for the first and second sets [0.25 (*n* = 521) and 0.26 (*n* = 546); *ps* < 0.001], while correlations between the single qualitative PDQ item and GDR were moderate for the first and second sets [0.41 (*n* = 459) and 0.45 (*n* = 481); *ps* < 0.001]. Finally, correlations between the PDQ Scale and HEI were significantly higher than correlations between the single qualitative PDQ item and HEI for the first set, but not the second set (*p* = 0.002 and 0.067). Correlations between the PDQ Scale and GDR were significantly higher than correlations between the single qualitative PDQ item and GDR for both sets (*p* < 0.001 and 0.036).

## 4. Discussion

We developed the PDQ Scale to measure the concept of perceived (self-assessed) diet quality. Thematic analysis of focus group discussions about rating one’s diet quality [15], existing questionnaire items, and a literature review of healthy eating concepts guided initial item development. We established content and face validity from nutrition experts and cognitive interviews with community residents. We conducted psychometric testing using data from the PDQ Study to examine the factor structure of the tool and to obtain evidence of validity and reliability. The results of the exploratory factor analysis revealed one factor for the PDQ Scale. The final PDQ Scale included seven items that measure an individual’s perception of the quality of their diet. We established concurrent and convergent validity through associations with the current single-item measure of PDQ and measured diet quality, respectively. While evidence for concurrent validity was strong, evidence for convergent validity was moderate to weak. However, correlations between measured diet quality indices and the PDQ Scale were generally significantly higher in magnitude than correlations between diet quality indices and the currently used single qualitative PDQ item.

In terms of factor structure and psychometric properties, placement (beginning vs. end of questionnaire) of the three self-assessed PDQ items yielded very similar results. The structure of the final factor solution was the same for both sets of items. Additionally, internal reliability, concurrent and convergent validity, and item response frequencies were similar between the two sets of items. Collectively, these results indicate that placement of the PDQ items in the questionnaire did not substantively affect responses, suggesting that earlier questions did not prime or alter how participants answered the later PDQ questions. In a study assessing question order for self-rated health, authors concluded that presenting the self-rated health question before domain-specific health items may increase inter-survey comparability because domain-specific health items vary across surveys [39]. This suggestion is applicable to the PDQ Scale as researchers may choose to use different measures to capture dietary intake.

Utility comparisons between the single-item PDQ and the new PDQ Scale are difficult as the new tool has yet to be used in studies to assess its associations with or predictive abilities for health measures beyond dietary intake. However, results from two studies assessing single versus multi-item self-rated health measures found that the multi-item measures performed better (e.g., more strongly associated with health outcomes) than the commonly used single questions [40,41]. Further, simulation research suggests that under most conditions typically encountered in practical applications, multi-item scales outperform single items in terms of predictive validity [42].

Future research includes collecting data from additional samples of community residents for further psychometric testing of the PDQ Scale (i.e., confirmatory factor analysis, divergent validity, and test–retest reliability). Additionally, determining if the PDQ Scale leads to more accurate assessment of one’s diet quality is needed to validate the use of the scale in place of the current single qualitative question.

The current study has strengths and limitations that bear mentioning. The sample size exceeded the target number of participants (*N* = 425) and thus was well powered to conduct psychometric testing. Collected data was self-reported and hence may be subject to bias. Participants were convenience samples of individuals residing in three states and thus results may not be generalizable to United States populations. However, the inclusion of participants from more than one state was a strength of the study. While the GDR was not designed to measure individual level diet quality, it was not used as a diagnostic or clinical tool in the current study. Additionally, the inclusion of more than one diet quality index is a strength of the study. The absence of confirmatory factor analysis, test–retest reliability assessment, and independent external validation represent limitations of the current study. Nonetheless, it is often challenging to address all components of instrument validation within a single research effort. The number of subject matter experts used to establish content validity was less than typically recommended; however, it exceeded the minimally acceptable number of 2 [43]. Additionally, although the review was not a formal, systematic content review, the experts provided meaningful confirmation that the items appropriately reflected the construct of perceived diet quality. Convergent validity for the PDQ Scale, while significantly higher than the single-item PDQ measure, was weak to moderate. Inclusion of three closely related PDQ items on the PDQ Scale may have superficially improved the scale’s reliability and construct validity. Detailed comparisons of the three items’ interrelationships and performance in terms of accurate assessment of diet quality is on-going and will be reported elsewhere. Because the PDQ Questionnaire was administered as an online survey, we took precautions to minimize, identify, and remove bot infection by checking for duplicate email addresses, telephone numbers, and first/last names and by calling individuals to verify valid telephone numbers, email addresses, and interest in study participation.

## 5. Conclusions

In conclusion, studying PDQ in today’s nutrition environment is critical given the growing emphasis on personalized nutrition, the widespread availability of nutrition misinformation, and the increasing reliance on self-directed dietary decision-making. Potential benefits of using a multi-item scale include a more comprehensive assessment of PDQ, increased sensitivity for detecting differences between individuals and change over time, and potentially higher reliability due to multiple indicators of PDQ. The proposed scale requires additional testing involving discriminant validity, test–retest reliability, and the determination of the predictive ability of the new tool for health outcomes before it can be recommended for research and clinical use. Given the additional testing, we anticipate that the PDQ Scale will be useful to health organizations, clinicians, researchers, and educators desiring evidence to guide the development of interventions to improve the accuracy of self-assessed diet quality. Ensuring that clinicians, researchers, and educators have a tool with strong psychometric properties to measure PDQ will improve the examination of the concept across settings, ultimately supporting more effective nutrition assessment and intervention efforts.

## Figures and Tables

**Table 1:** Perceived Diet Quality Questionnaire items.

Item	Responses	Source
In general, how healthy is your diet?	Excellent	[18]
	Very good	
	Good	
	Fair	
	Poor	
How would you rate the quality (healthfulness) of your diet? Enter a number between 1 (horrible) to 10 (perfect). ^1^	1–10	[15]
Think of letter grades, like in school, how would you “grade” the quality (healthfulness) of your diet? ^1^	A	[15]
	B	
	C	
	D	
	F	
How satisfied are you with the quality (healthfulness) of your diet? ^1^	Very satisfied	[15]
	Satisfied	
	Neither satisfied nor dissatisfied	
	Dissatisfied	
	Very dissatisfied	
How often do you feel your meals are balanced? By balanced, we mean containing a variety of fruits, vegetables, whole grains, protein, and dairy. ^1^	Always	[15,19]
	Often	
	Sometimes	
	Rarely	
	Never	
How often do you plan your meals in advance (e.g., meal prep)? ^1^	Always	[15]
	Often	
	Sometimes	
	Rarely	
	Never	
How often do you cook meals at home versus eating out or ordering takeout? ^1^	All meals cooked at home	[15]
	Most meals cooked at home	
	About the same between meals	
	cooked at home and eating	
	out/takeout	
	Most meals eaten out/takeout	
	All meals eaten out/takeout	
I feel I have a good knowledge of nutrition.	Strongly agree	[20]
	Agree	
	Neither agree nor disagree	
	Disagree	
	Strongly disagree	
Do you use information on food labels when deciding what foods to buy or eat?	Always	[18]
	Often	
	Sometimes	
	Rarely	
	Never	
Do you find it hard to eat healthy? ^1^	Yes—always	[15]
	Yes—sometimes	
	No	
How often do you feel guilty or regretful about your food choices? ^1^	Always	[15]
	Often	
	Sometimes	
	Rarely	
	Never	

^1^ Item based on results from thematic analysis of focus group discussions.

**Table 2:** PDQ Study participant characteristics (*N* = 605).

Characteristic	*n*	%
Age, years		
18–19	41	6.8
20–29	144	23.9
30–39	158	26.2
40–49	109	18.1
50–59	75	12.5
60–69	62	10.3
≥70	12	2.0
Gender		
Male	172	28.8
Female	425	71.1
Relationship status		
Married	186	31.1
Widowed	29	4.8
Divorced	37	6.2
Separated	44	7.3
Never married	199	33.2
Living with partner	64	10.7
Education level		
Some high school or less	27	4.5
High school diploma or GED	151	25.2
Some college but not degree	106	17.7
Associate or technical degree	104	17.3
Bachelor’s degree	124	20.7
Graduate or professional degree	74	12.3
Work status		
Employed full time (≥30 h/week)	316	52.4
Employed part time (<30 h/week)	92	15.3
Not employed and looking for work	67	11.1
Not employed and not looking for work	21	3.5
Retired	41	6.8
Disabled and not able to work	42	7.0
Hispanic/Latino/Spanish		
Yes	32	6.0
No	484	91.1
Race		
American Indian or Alaska Native	15	2.6
Asian	6	1.0
Black or African American	387	66.6
Native Hawaiian or Pacific Islander	3	0.5
White	161	27.7
Other	9	1.5
Income		
≤$25,000	133	22.7
$25,001–$35,000	99	16.9
$35,001–$45,000	70	12.0
$45,001–$55,000	54	9.2
$55,001–$65,000	42	7.2
$65,001–$75,000	47	8.0
$75,001–$85,000	26	4.4
$85,001–$95,000	12	2.1
$95,001–$105,000	14	2.4
$105,001–$115,000	14	2.4
$115,001–$125,000	5	0.9
≥$125,001	6	1.0

PDQ, Perceived diet quality; GED, General Education Development exams.

**Table 3:** Factor structure of the Perceived Diet Quality Questionnaire.

	1st Diet Quality Indices (*n* = 573)			2nd Diet Quality Indices (*n* = 580)		
Item	F1 ^1^	F2 ^1^	CV	F1 ^1^	F2 ^1^	CV
	Initial factor solution (2 factors)					
PDQ graded	0.84	0.06	0.74	0.82	0.11	0.73
PDQ qualitative	0.83	0.02	0.70	0.85	0.02	0.74
Balanced meals	0.83	−0.08	0.66	0.85	−0.10	0.68
Satisfaction with diet healthfulness	0.77	0.07	0.63	0.74	0.07	0.59
PDQ numeric	0.74	0.14	0.63	0.76	0.18	0.70
Meal planning/preparation	0.67	−0.01	0.45	0.69	−0.02	0.47
Nutrition knowledge	0.64	0.02	0.42	0.67	0.01	0.45
Food label use	0.64	−0.14	0.38	0.66	−0.16	0.40
Home cooking vs. eating out/takeout	0.43	0.10	0.22	0.45	0.10	0.24
Food choice guilt/regret	−0.10	0.87	0.72	−0.11	0.89	0.74
Difficulty eating healthy	0.25	0.46	0.34	0.25	0.45	0.33
	Final factor solution (1 factor)					
PDQ graded	0.88	--	0.78	0.87	--	0.76
PDQ qualitative	0.86	--	0.74	0.87	--	0.75
Satisfaction with diet healthfulness	0.81	--	0.65	0.78	--	0.61
PDQ numeric	0.80	--	0.64	0.82	--	0.67
Balanced meals	0.79	--	0.62	0.80	--	0.65
Meal planning/preparation	0.64	--	0.40	0.66	--	0.44
Nutrition knowledge	0.62	--	0.38	0.65	--	0.43

F1, factor 1; F2, factor 2; CV, communality value; PDQ, perceived diet quality. ^1^ Pattern coefficients (factor loadings).

## Data Availability

The original data presented in the study are openly available in USDA Ag Data Commons at https://doi.org/10.15482/USDA.ADC/32207928.v1.

## Abbreviations

The following abbreviations are used in this manuscript:

DQQ	Diet Quality Questionnaire
GDRs	Global Dietary Recommendations
HEI	Healthy Eating Index
PDQ	Perceived diet quality
USDA	United States Department of Agriculture
